# Soil Fungal Diversity, Community Structure, and Network Stability in the Southwestern Tibetan Plateau

**DOI:** 10.3390/jof11050389

**Published:** 2025-05-19

**Authors:** Shiqi Zhang, Zhenjiao Cao, Siyi Liu, Zhipeng Hao, Xin Zhang, Guoxin Sun, Yuan Ge, Limei Zhang, Baodong Chen

**Affiliations:** 1 State Key Laboratory of Regional and Urban Ecology, Research Center for Eco-Environmental Sciences, Chinese Academy of Sciences, Beijing 100085, Chinacaozhenjiao@ibcas.ac.cn (Z.C.); syliu@rcees.ac.cn (S.L.);; 2 University of Chinese Academy of Sciences, Beijing 100049, China

**Keywords:** Tibetan Plateau, fungal diversity, biogeography, influencing factors

## Abstract

Despite substantial research on how environmental factors affect fungal diversity, the mechanisms shaping regional-scale diversity patterns remain poorly understood. This study employed ITS high-throughput sequencing to evaluate soil fungal diversity, community composition, and co-occurrence networks across alpine meadows, desert steppes, and alpine shrublands in the southwestern Tibetan Plateau. We found significantly higher fungal α-diversity in alpine meadows and desert steppes than in alpine shrublands. Random forest and CAP analyses identified the mean annual temperature (MAT) and normalized difference vegetation index (NDVI) as major ecological drivers. Mantel tests revealed that soil physicochemical properties explained more variation than climate, indicating an indirect climatic influence via soil characteristics. Distance–decay relationships suggested that environmental heterogeneity and species interactions drive community isolation. Structural equation modeling confirmed that the MAT and NDVI regulate soil pH and carbon/nitrogen availability, thereby influencing fungal richness. The highly modular fungal co-occurrence network depended on key nodes for connectivity. Vegetation coverage correlated positively with network structure, while soil pH strongly affected network stability. Spatial heterogeneity constrained stability and diversity through resource distribution and niche segregation, whereas stable networks concentrated resources among dominant species. These findings enhance our understanding of fungal assemblage processes at a regional scale, providing a scientific basis for the management of soil fungal resources in plateau ecosystems.

## 1. Introduction

Soil fungi are key drivers of terrestrial ecosystem processes, maintaining ecosystem productivity and stability through organic matter decomposition [1], nutrient cycling [2], and symbiotic relationships with plants (e.g., mycorrhizal fungi) [3], as well as improving soil structure, suppressing phytopathogens, stabilizing edaphic trophic networks [4], sequestering carbon [2], and degrading chemical contaminants [5]. In recent years, the application of high-throughput sequencing technology has significantly expanded our understanding of fungal diversity, distribution, and functional networks [6]. Global-scale studies indicate that climate factors (e.g., temperature and precipitation) primarily regulate fungal community distribution by influencing resource availability [1], whereas regional-scale analyses emphasize the synergistic effects of soil physicochemical properties (e.g., pH, carbon, and nitrogen content) and plant communities [7,8]. For example, in temperate forests, the heterogeneity of plant litter promotes the functional differentiation of saprotrophic fungi [9], while in arid ecosystems, water limitation alters community structure by restricting the colonization of symbiotic fungi [10]. Chen et al. [11] found that ammonium nitrogen scarcity may inhibit symbiotic fungi colonization in alpine ecosystems, while Cui et al. [12] observed that the total soil carbon content promotes fungal diversity by improving microenvironments at the local scale. However, this effect is masked by precipitation gradients in cross-climate zone analyses. These findings suggest that fungal community distribution results from the combined effects of climate, soil, and vegetation interactions, with the relative importance of these drivers varying across spatial scales and ecosystem types.

Although previous studies have highlighted the roles of climate, soil, and vegetation in fungal distribution, several gaps remain. Current research primarily focuses on temperate or low-elevation ecosystems, with limited studies on fungal community adaptations to alpine and arid environments [11]. Additionally, although many global- and regional-scale studies on fungal distributions exist, systematic investigations across diverse habitats that address how multiple environmental factors affect fungal network structures are comparatively rare.

The structural features of soil fungal co-occurrence networks, such as connectivity, modularity, and centrality, are crucial indicators of community stability [13], while network complexity and modularity are considered essential for maintaining ecosystem functions, especially key services such as carbon cycling and nutrient transformation [13]. In fungal co-occurrence networks, highly connected networks enhance system stability through resource exchange or collaborative degradation functions between species, helping communities to resist environmental pressures [14]. Modularity maintains ecosystem diversity and stability by dividing community functions into different modules (such as decomposition, symbiosis, and pathogenic modules). However, environmental pressures such as climate change and drought may disrupt the balance of resource distribution, leading to network collapse or functional loss [15]. Under drought stress, fungal networks may prioritize core functional groups by reducing inter-module connections, but this restructuring often leads to a loss of species diversity [15]. Understanding how fungal network structures respond to environmental gradients is crucial not only for understanding ecosystem stability, but also for providing theoretical support for microbial resource conservation strategies.

The Tibetan Plateau, one of the highest and fastest-warming regions in the world, offers a natural laboratory for studying the biogeographical patterns of fungal communities [16]. Although previous studies have shown that fungal diversity in this region is significantly lower than that in low-elevation ecosystems [17,18], the driving factors behind the spatial heterogeneity of fungal diversity remain controversial. Some studies suggest that low temperature is the primary factor limiting community assembly [9,19], while others emphasize the critical role of soil nutrient availability and plant interactions [20,21]. Furthermore, rapid climate change may exacerbate the vulnerability of fungal network structures by altering water–heat balance and freeze–thaw cycles [15]. However, existing regional studies are mostly limited to a single vegetation type, lacking systematic analysis across spatial scales and vegetation types, and failing to reveal how multidimensional environmental pressures influence fungal network stability [22]. This gap has led to the long-term “black-box” status of developing strategies for maintaining and regulating fungal communities in alpine ecosystems. In complex terrain areas like the Tibetan Plateau, adjacent sampling points may span multiple habitat types, such as desert steppes and alpine meadows, creating unique habitat mosaics. Abrupt water–heat changes in these habitats may strengthen environmental filtering effects, leading to rapid turnover of functional groups (such as transitions between saprotrophic and symbiotic fungi), while fragmented habitat patches may reconstruct species interaction networks through habitat selection, forming stability mechanisms that are distinct from continuous gradient zones [13]. However, the spatial differentiation patterns of fungal diversity and the evolutionary paths of cross-habitat network topology in such mixed habitats have yet to be systematically analyzed.

By integrating cross-habitat fungal community data in the southwestern Tibetan Plateau, the present study systematically analyzes the fungal diversity differentiation, network stability maintenance mechanisms, and spatial coupling effects of the climate, soil, and vegetation. We aim to address the following scientific questions: First, do soil fungal communities exhibit cross-habitat divergence along environmental gradients, and what specific climatic, edaphic, or vegetative factors drive fungal spatial distribution patterns? Second, within each habitat, which environmental variables predominantly shape the size, connectivity, and modularity of the fungal co-occurrence network, and how do key nodes—such as module hubs and connectors—underpin overall network stability and adaptation to environmental stress? Third, how does landscape-scale spatial heterogeneity—through its effects on the local climate and vegetation productivity—alter soil physicochemical properties, and thereby regulate fungal diversity and network resilience, in these high-altitude ecosystems?

## 2. Materials and Methods

### 2.1. Study Area and Soil Sampling

**Figure 1:**
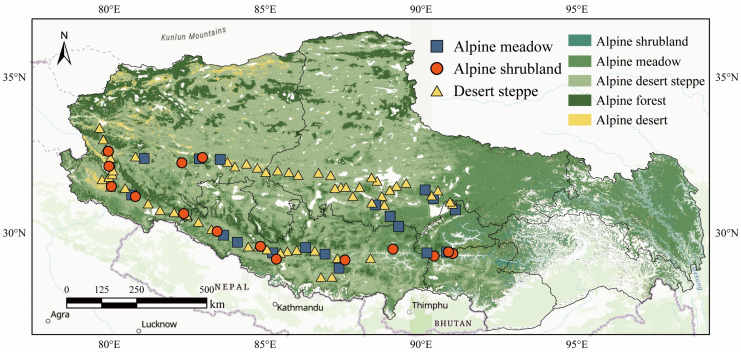
The distribution of sampling sites in the southwestern Tibetan Plateau.

The study area is in the southwestern part of Tibet, with a geographical span of 1700 km, covering latitudes from 28.31° N to 33.53° N and longitudes from 79.69° E to 91.29° E. The altitude ranges from 3570 m to 5745 m. Three distinct ecological types—alpine meadows, desert steppes, and alpine shrublands—were surveyed at 92 sites (33 in alpine meadows, 42 in desert steppes, and 17 in alpine shrublands). Sites were distributed at roughly 20–25 km intervals across the study area, with the habitat type confirmed onsite by assessing vegetation composition and soil texture. Soil samples from alpine shrublands were collected in July–August 2020, whereas samples from alpine meadows and desert steppes were collected in July–August 2021 (Figure 1). This period is the peak growing season, when the temperature and moisture are stable, microbial activity is the highest, and snow cover is absent; thus, the selection of this period ensured consistent, comparable sampling across all habitats. At each sampling point, three replicate quadrats were randomly selected, with a distance of over 30 m between quadrats. Important geographical variables, such as longitude, latitude, altitude, and slope, were recorded. Vegetation cover was assessed, and variables including species richness, dominant species, and community composition were recorded for each quadrat. Aboveground biomass was quantified using mowing techniques, and samples were packed into envelopes for further analysis. Within each plot, five soil cores (0–10 cm depth) were collected according to a five-point pattern, combined, and quartered to yield approximately 600 g of fresh composite soil per plot; these samples were rapidly sealed in self-sealing bags and stored in portable coolers. After transportation to the lab, all soil samples were sieved through a 2 mm mesh, and visible stones and roots were removed. Then, each sample was divided into two portions: one was air-dried for subsequent physical and chemical analyses, and the other was stored at −20 °C for microbial analysis.

### 2.2. Climate Parameters and Soil Physicochemical Properties

Climate-associated parameters, such as the mean annual temperature (MAT) and mean annual precipitation (MAP), were ascertained for each sampling locale by Kriging interpolation techniques. These assessments drew upon a dataset capturing a decadal average from 2010 to 2020 for both temperature and precipitation, provided by the National Meteorological Information Centre (http://data.cma.cn/en, accessed on 24 August 2024).

The soil total nitrogen content was ascertained using an elemental analyzer (Vario MAX cube, Elementar, Langenselbold, Germany) [23], and the content of soil organic carbon was determined by the dichromate oxidation method, applied to air-dried soil [24]. A mixture of soil and deionized water, at a ratio of 1:2.5 (soil:water, *w*:*v*), was prepared, and the soil pH was subsequently measured using a pH meter [25]. To quantify the concentrations of ammonium nitrogen and nitrate nitrogen in the soil, 10 g of air-dried soil was exposed to 50 mL of 2 mol L^−1^ KCl extract, shaken at 180 rpm for 1 h, and filtered with medium-speed qualitative filter paper [26]. This filtrate was then subjected to analysis with a continuous flow analyzer (AA3, Seal, Hamburg, Germany). Lastly, the sodium bicarbonate (NaHCO_3_, pH = 8.5) molybdenum antimony anti-colorimetric method was employed to evaluate the available phosphorus in the soil [27].

### 2.3. DNA Extraction and High-Throughput Amplicon Sequencing

Soil sample DNA was isolated using the FastDNA^TM^ SPIN Kit for Soil (MP Biomedicals, Burlingame, CA, USA), along with the FastPrep-24^TM^ 5G sample disruption instrument. Quantification of the extracted DNA was performed using an ultra-micro spectrophotometer (NanoDrop1000, Thermo Fisher Scientific, Waltham, MA, USA). The ITS2 region of the soil fungus underwent PCR amplification with gITS7ngs/ITS4ngsUni primers. The resulting products were purified, a library was constructed, and paired-end sequencing was carried out on the Illumina HiSeq 2500 high-throughput sequencing platform (Illumina, San Diego, CA, USA) in PE 250 mode. The bioinformatic analysis of the sequenced data involved the DADA2 (Divisive Amplicon Denoising Algorithm, v.1.20.0) sequence denoising algorithm. The initial step was the removal of primers at both sequence ends using Cutadapt v3.4 (https://cutadapt.readthedocs.io/, accessed on 6 February 2023), followed by the filterAndTrim function in the DADA2 package v1.20.0 to eliminate low-quality sequences for data quality control. Subsequently, the learnErrors and dada functions were applied to assess the error rate and infer amplicon sequence variant (ASV) sequences. The mergePairs function then concatenated the forward and reverse sequences, while the removeBimeraDenovo function eliminated chimeric sequences, culminating in the generation of an ASV table. The concluding step involved species annotation of the resulting ASV sequences utilizing the naive Bayesian classifier method, according to the UNITE fungal database. This process led to the identification of a total of 8788 fungal ASVs from the 276 soil samples in this study.

### 2.4. Statistical Analysis

The soil fungal ASV richness index was calculated using the R package “vegan” (vegan 2.6–4), and random forest (RF) analysis was performed to assess the role of soil and climate variables as predictors of fungal richness. To assess the significance and importance of environmental variables on fungal β-diversity, distance-based redundancy analysis (db-RDA) and forward selection using Bray–Curtis distance matrices were employed to estimate the proportion of explained variance (R^2^). The forward selection stopped when the significance level was α (*p* > 0.05), or when adding a new variable did not significantly increase the explained variance (R^2^). The results were visualized using constrained analysis of principal coordinates (CAP), revealing the impact of environmental variables on fungal community structure. To further assess the importance of climate and soil variables, standard and partial Mantel tests were performed using the “vegan” package v2.6.10 and “geosphere” package v1.5.18, and spatial distance–decay relationships of community dissimilarity were evaluated. Regression analysis was conducted using the Levenberg–Marquardt (L-M) method to quantify the relationships between variables and predict their trends. All data analyses were performed in R-4.2.2. Generalized Additive Models (GAMs) were employed to explore the relationship between environmental factors and fungal community structure. GAMs effectively capture the nonlinear effects of variables, which is crucial for revealing the complex and dynamic impacts of environmental factors, including temperature, precipitation, soil nutrients, and vegetation cover, on fungal diversity and functional group distribution.

### 2.5. Network Structure Analysis

For network construction, only ASVs with relative abundances greater than 0.01% of the total fungal sequences were retained. Spearman correlations and Kullback–Leibler divergence (KLD) between ASVs were calculated, with a Spearman correlation threshold of 0.6, and the maximum KLD matrix value set as the divergence threshold [28]. To ensure reliability, 1000 resamplings were performed, and *p*-values were generated to assess the statistical significance of association metrics. Adjusted *p*-values were corrected using the Benjamini–Hochberg method [29], and only network edges with Spearman correlations and KLD values meeting the criteria and corrected *p*-values < 0.05 were retained. A total of 276 soil samples were analyzed across the three ecosystem types. From the global OTU co-occurrence network, we generated one subnetwork per sample by extracting the induced subgraph of the taxa presenting in that sample using the induced_subgraph function in the igraph package v1.4.2. For each sample-specific subnetwork, we then calculated topological metrics, including the node count, edge count, and average path length. Module analysis was used to identify key modules in the network, and nodes were classified according to their connectivity within the module (Zi) and between modules (Pi), based on the method of Shi et al. [30]. Nodes were categorized as module hubs (highly connected within a module, Zi > 2.5), network hubs (highly connected in the entire network, Zi > 2.5 and Pi > 0.62), connectors (nodes linking modules, Pi > 0.62), and peripherals (nodes with few external connections, Zi < 2.5 and Pi < 0). Module hubs, network hubs, and connectors were considered as key nodes, due to their important role in microbial community structure and potential functions. The method described by Fu et al. [31] was applied to calculate the fungal network stability based on the ratio between negative and positive cohesions in the network.

### 2.6. Multidimensional Scaling (MDS) and Structural Equation Modeling (SEM)

To quantify the impact of geographic distance on the fungal ecological network, we calculated a Euclidean distance matrix based on the geographic coordinates of sampling sites and extracted spatial axes (MEM axes) using multidimensional scaling (MDS) analysis [32]. The axes generated by the MDS analysis represent the relative spatial relationships among samples, with the first axis typically reflecting the primary spatial gradient. The cumulative explanation rate indicated that the first axis accounted for 94% of the variance in spatial distance; thus, we selected the first axis as the spatial factor. To ensure consistency with other environmental factors, the axis coordinates were standardized using Z-score transformation. All analyses were performed in R using the cmdscale function.

A structural equation model (SEM) was built using AMOS v26.0 to verify the direct or indirect effects of spatial factors, climate factors, vegetation (NDVI), and soil properties on soil fungal α-diversity and network stability. It was hypothesized that spatial factors, climatic factors, vegetation, and soil properties would collectively and significantly influence fungal α-diversity and network stability. Spatial factors not only directly impact fungal diversity and network stability, but also indirectly affect soil properties by regulating the MAT and NDVI. Soil properties (e.g., pH, total carbon, and nitrate nitrogen) further significantly influence fungal diversity and network stability, thereby establishing a multi-level regulatory mechanism. The initial SEM included all reasonable pathways, and the model was optimized by stepwise removal of non-significant paths (*p* > 0.05). Model fit was evaluated using fit indices, including chi-square tests (*p* > 0.05), RMSEA (<0.08), TLI (>0.90), and CFI (>0.95).

## 3. Results

### 3.1. The Impact of Environmental Factors on Soil Fungal Communities

**Figure 2:**
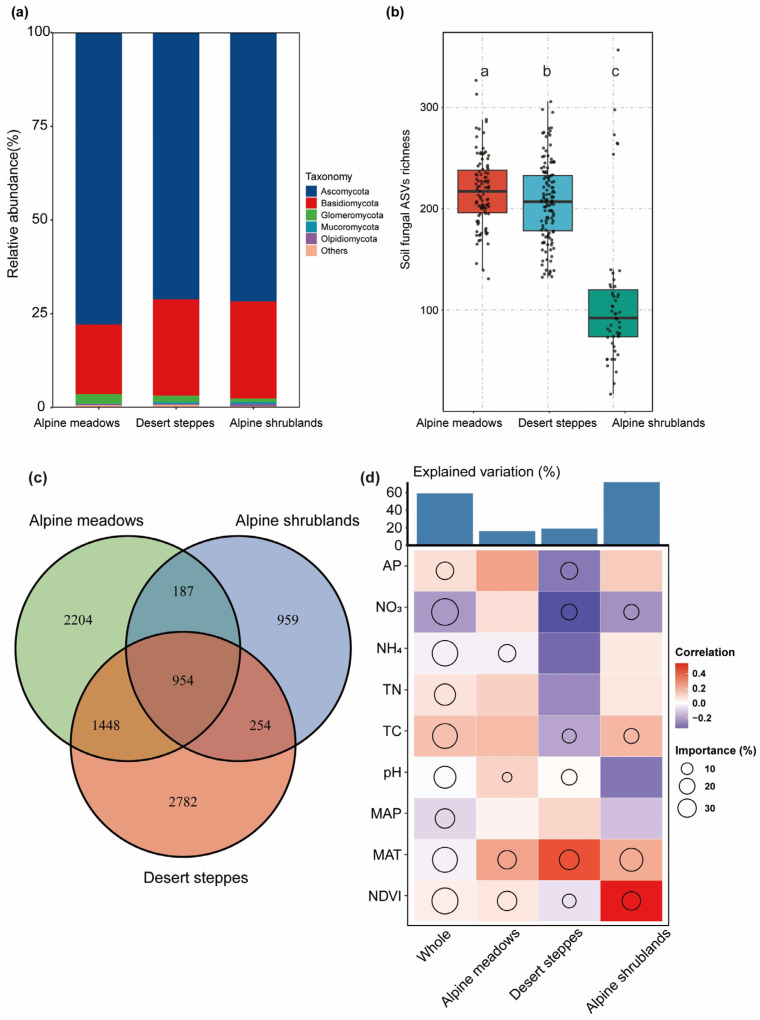
Soil fungal community composition, diversity, and environmental drivers across habitats. (**a**) Soil fungal community composition in alpine meadows, desert steppes, and shrublands (classified by phylum). (**b**) Differences in soil fungal diversity among alpine meadows, desert steppes, and shrublands. Different letters (a, b, c) above the bars indicate statistically significant differences among habitats based on one-way ANOVA followed by Tukey’s HSD post hoc test (*p* < 0.05). (**c**) A Venn diagram showing the number of unique and overlapping ASVs. (**d**) Contributions of environmental factors to fungal richness, based on correlations and random forest models. Circle size represents variable importance (the percentage increase in mean squared error, calculated using the random forest model). Colors indicate Spearman correlation coefficients.

In the alpine meadow, desert steppe, and alpine shrubland habitats, Ascomycota was unexceptionally the dominant fungal phylum, and the relative abundance of Ascomycota was consistently higher than that of other fungal groups (Figure 2a). The number of ASVs detected varied among the three habitats: 5438 ASVs in alpine meadows, 4793 ASVs in desert steppe, and 2354 ASVs in alpine shrublands (Figure 2c). Additionally, the number of unique ASVs detected in each individual ecosystem was 2204 in alpine meadows, 2782 in desert steppes, and 959 in alpine shrublands. A total of 954 ASVs were shared among the three habitats. Regarding functional groups, the number of saprotrophic fungi in alpine meadows and desert steppes was significantly higher than that in alpine shrublands, while the number of symbiotic fungi in desert steppes was significantly higher than that in alpine shrublands. However, no significant differences were observed for pathogenic fungi among the different habitats (Figure S1).

The soil fungal α-diversity showed significant differences between habitats (Figure 2b). In terms of ASV richness, there was a significant difference for alpine meadows and desert steppes; both habitats had a significantly higher ASV richness than alpine shrublands.

To identify the main environmental variables influencing α-diversity, a random forest analysis was performed. The results showed that the MAT and NDVI contributed the most to explaining the variation in fungal richness across habitats (Figure 2d). Among all biotic and abiotic factors, the NDVI (*r*^2^ = 0.06, *p* < 0.001), MAT (*r*^2^ = 0.06, *p* < 0.001), TC (*r*^2^ = 0.02, *p* < 0.05), and NO_3_^−^ (*r*^2^ = 0.07, *p* < 0.01) were significantly correlated with fungal species richness, while the MAP, pH, TN, NH_4_^+^, and AP showed no significant correlations (Figure S2a–i).

**Figure 3:**
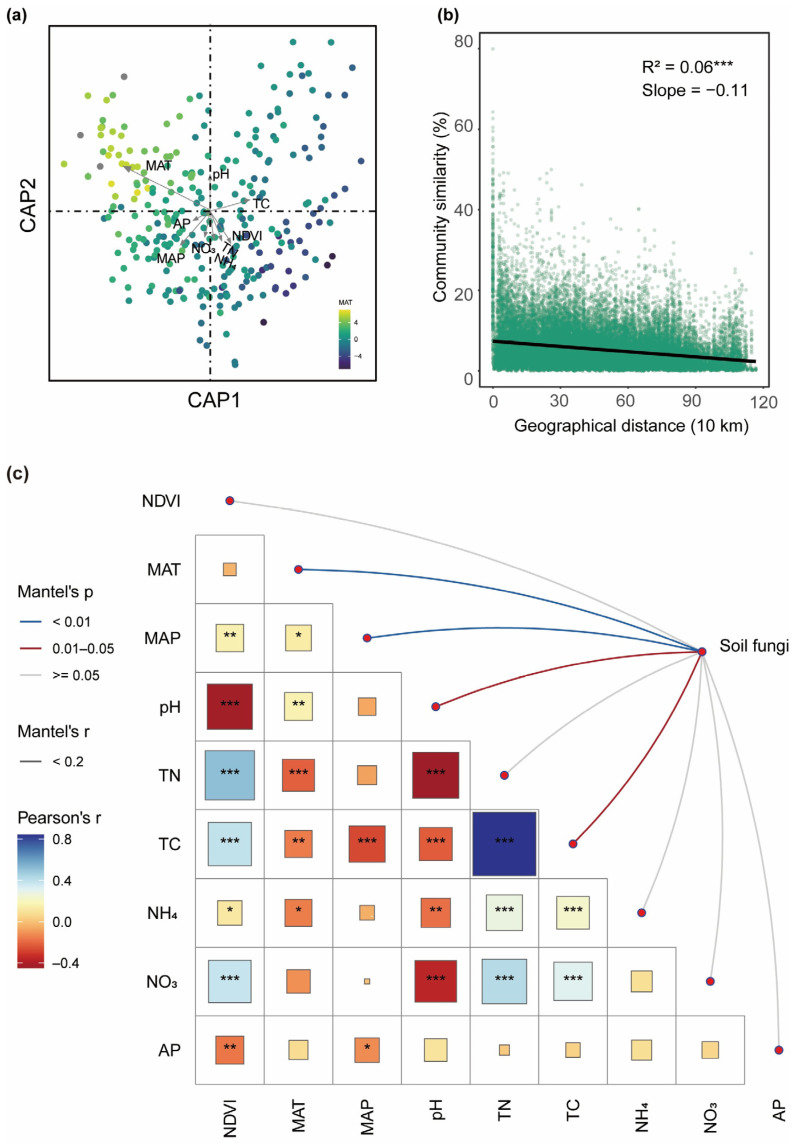
Environmental drivers and geographical patterns of soil fungal communities. (**a**) Constrained analysis of principal coordinates (CAP) showing the influence of environmental factors on fungal community structure. Sample points are colored according to MAT (mean annual temperature). (**b**) Distance–decay curves showing Bray–Curtis similarity as a function of geographical distance between sampling points. The solid line represents ordinary least-squares linear regression. (**c**) Environmental drivers of soil fungal communities evaluated through Mantel tests. MAT, mean annual temperature; MAP, mean annual precipitation; NDVI, normalized difference vegetation index; TN, soil total nitrogen content; TC, soil total carbon content; NH_4_, soil ammonium nitrogen content; NO_3_, soil nitrate nitrogen content; AP, soil available phosphorus content. Asterisks indicate significant correlations (*, *p* < 0.05; **, *p* < 0.01; ***, *p* < 0.001).

Additionally, CAP analysis was used to identify the key environmental variables influencing fungal β-diversity (Figure 3a,c). The MAT was identified as the most important predictor of fungal β-diversity across all habitats (Figure 3a and Figure S3a, Tables S1–S4). Mantel and partial Mantel tests quantified the contributions of soil and climatic variables to β-diversity (Table S5). When controlling soil variables, the correlation between climatic variables and fungal β-diversity significantly decreased, particularly in desert steppes and alpine shrublands, indicating that the independent explanatory power of climatic variables was weaker. Although the MAT showed a substantial impact on β-diversity, the influence of soil variables was more pronounced. Climatic variables affected fungal communities indirectly through regulation of soil variables. Moreover, the relationship between community similarity and geographic distance for each sample pair exhibited significant distance–decay (slope = −0.11) (Figure 3b).

### 3.2. Fungal Network Structure and Ecological Function

**Figure 4:**
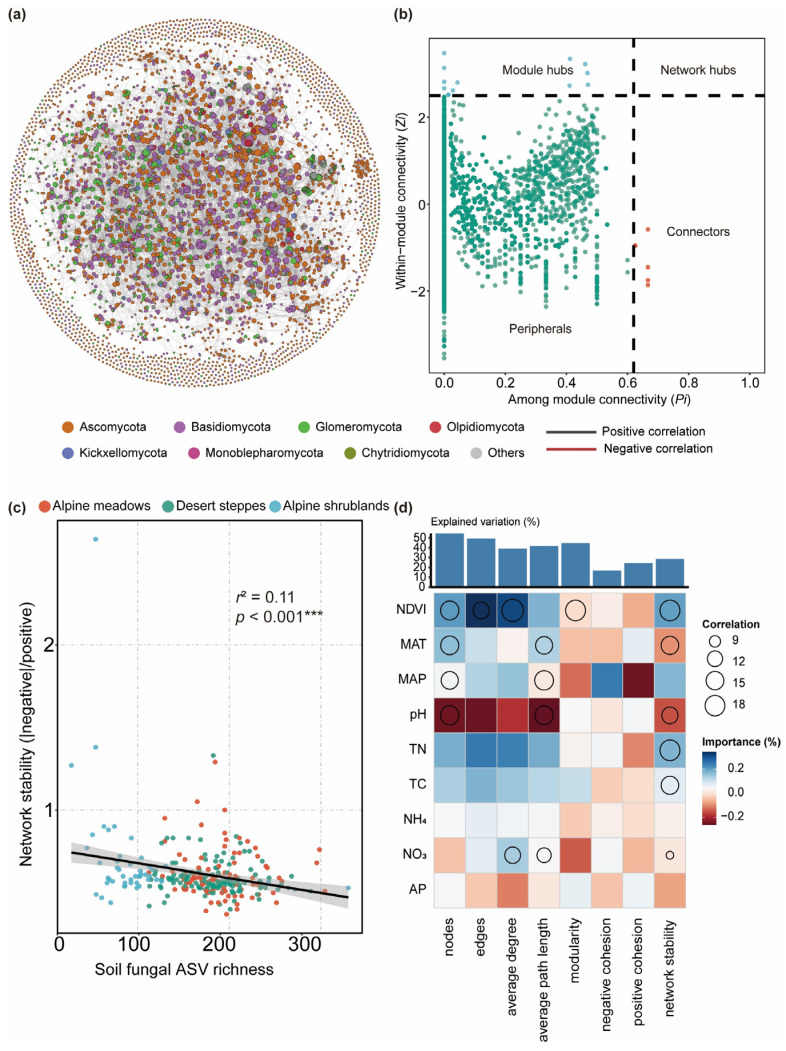
Co-occurrence networks, keystone species, and environmental influences on soil fungal community stability. (**a**) Soil fungal co-occurrence network: nodes represent ASVs colored by phylum; edge color indicates positive (black) or negative (red) correlations, and thickness indicates their strength. (**b**) Keystone species analysis of soil fungal communities. Based on Zi (within-module connectivity) and Pi (among-module connectivity) values, network nodes are classified into four categories: network hubs (Zi > 2.5, Pi > 0.62) represent ASVs that are highly connected both overall and within modules; module hubs (Zi > 2.5, Pi < 0.62; blue dots) are ASVs highly connected only within one module; ASVs linking modules are termed connectors (Zi < 2.5, Pi > 0.62; orange dots); and peripherals (Zi < 2.5, Pi < 0.62; green dots) have few connections with other species. (**c**) The relationship between network stability and soil fungal ASV richness index. Network stability is calculated as the absolute difference between negative and positive cohesions. *** indicates a highly significant correlation (*p* < 0.001). (**d**) Contributions of environmental factors to fungal network structure and stability based on correlations and random forest models. Circle size represents variable importance (percentage increase in mean squared error, calculated using the random forest model). Colors indicate Spearman correlation coefficients.

At the regional scale, fungal community networks exhibit a highly modular structure (modularity index = 0.879), consisting of 7254 nodes and 67,578 edges, with an average of 18.63 connections per node (Figure 4a). Through Zi-Pi analysis, two types of key nodes were identified in the network: module hubs and connectors. A total of 15 module hubs were identified, 12 of which belonged to the phylum *Ascomycota*, with saprotrophs as the dominant functional type (Figure 4b, Table S6). These nodes exhibited high connectivity within modules, playing a critical role in maintaining the structural stability of local modules. Seven connector nodes were identified, distributed across *Ascomycota*, *Glomeromycota*, and *Basidiomycota*, primarily enhancing the overall connectivity and adaptability of the network through the flow of resources and information between modules. Saprotrophs dominated the network, accounting for 70.91% of all nodes, highlighting their central role in decomposition. Symbiotrophs accounted for 19.33%, and pathotrophs accounted for 9.8%. Despite their lower proportions, they played important roles in specific modules.

Environmental variables significantly influenced the structure and function of fungal co-occurrence networks. Mantel tests showed that the NDVI was significantly positively correlated with the number of nodes (*r* = 0.131, *p* < 0.001), the number of edges (*r* = 0.266, *p* < 0.001), and the average degree (*r* = 0.191, *p* < 0.001), indicating that vegetation coverage promotes the functionality of modules and key nodes by enhancing network size and connectivity (Table S7). The MAT had a weaker positive effect on the number of nodes (*r* = 0.076, *p* < 0.05) and edges (*r* = 0.078, *p* < 0.05). Soil pH was significantly positively correlated with the number of nodes (*r* = 0.060, *p* < 0.05), the number of edges (*r* = 0.063, *p* < 0.05), and the average path length (*r* = 0.094, *p* < 0.01), indicating its important role in influencing network stability by regulating module distribution (Table S7).

Total nitrogen (TN) was significantly positively correlated with the node count (*r* = 0.09, *p* = 0.02), edge count (*r* = 0.19, *p* = 0.01), and average degree (*r* = 0.14, *p* = 0.01), indicating that sufficient nitrogen supply may provide resource support for fungal networks (Table S7). Additionally, TN was significantly correlated with the mean path length (*r* = 0.09, *p* = 0.02). Total carbon (TC) showed a positive correlation with the node count (*r* = 0.07, *p* = 0.05), edge count (*r* = 0.14, *p* = 0.02), and average degree (*r* = 0.10, *p* = 0.02), and was significantly correlated with the mean path length (*r* = 0.10, *p* = 0.01), suggesting that carbon resources positively influence network size and connectivity. In contrast, the MAP, NH_4_^+^, and AP had minimal direct effects on network properties. The MAP was not significantly correlated with any network property (*p* > 0.05), and NH_4_^+^ and AP showed no significant effects on the number of nodes, the number of edges, or the modularity index (*p* > 0.05). NO_3_^−^ exhibited marginal significance for the number of edges (*r* = 0.070, *p* = 0.074) and average degree (*r* = 0.069, *p* = 0.063) (Table S7).

Subnetwork analysis for each sampling site indicated that the structure and stability of fungal networks varied across habitats (Figure S4). Alpine meadows had the highest node number, edge number, and average degree, suggesting stronger species interactions (Figure S4a–c). The average path length was longest in alpine meadows, whereas it was shorter in desert steppes and alpine shrublands, with no significant difference between the two (Figure S4d). This suggests that the meadow fungal network has more indirect connections, potentially reducing resources and information transmission efficiency. However, the global clustering coefficient was significantly higher in alpine meadows than in desert steppes and alpine shrublands, indicating stronger local clustering (Figure S4e), which promotes resource sharing and cooperative interactions. In contrast, alpine shrubland exhibited the highest modularity. Network stability (cohesion) and fungal α-diversity (richness) showed a significant negative correlation (*r*^2^ = 0.11, *p* < 0.001) (Figure 4c), suggesting that network stability may restrict community diversity expansion under certain environmental conditions by enhancing the functionality of key nodes and modules.

Random forest analysis further revealed the regulatory effects of environmental factors on network structure and stability (Figure 4d). The NDVI was significantly important for the node count, edge count, average degree, modularity, and network stability (*p* < 0.01), indicating that vegetation coverage levels significantly enhance network stability by regulating interactions among fungal communities (Table S7). The MAT and MAP significantly affected the average path length (*p* < 0.01), indicating the importance of climatic factors in the efficiency of information and resource transmission within the network (Table S7). The soil pH significantly influenced network properties, including the node count, average path length, and network stability (*p* < 0.01), suggesting that pH could further affect network size and structure by regulating fungal ecological adaptation (Table S7). Additionally, TN and TC significantly impacted network stability (*p* < 0.01). In contrast, NH_4_^+^ and AP had no significant effects on most network properties (*p* > 0.05), suggesting their weak direct regulatory role and likely dependence on indirect effects from other environmental factors (Figure 4d, Table S7).

### 3.3. Influencing Factors for Fungal Diversity and Network Stability

Using Generalized Additive Models (GAMs) to analyze the environmental drivers of fungal network stability across different habitats (Table S8), we found that in alpine meadows, fungal network stability is nonlinearly regulated by various environmental factors, with a strong model fit (adjusted *R*^2^ = 0.736, deviance explained = 80.1%). Specifically, the NDVI (*p* < 0.001), MAT (*p* < 0.001), soil pH (*p* < 0.001), and NO_3_^−^ (*p* = 0.004) have significant nonlinear positive effects on network stability. In contrast, TN (*p* = 0.315), TC (*p* = 0.201), and NH_4_^+^ (*p* = 0.052) have no significant effects.

In desert steppes, the environmental factors influencing fungal network stability are simpler, and the model fit is weaker (adjusted *R*^2^ = 0.242). The NDVI (*p* = 0.001) and MAT (*p* = 0.048) are significantly positively correlated with network stability. Additionally, AP (*p* = 0.003) also has a significant linear positive effect on network stability. Other factors, such as TN, TC, and soil pH, do not significantly affect network stability, suggesting that the network stability in this habitat is primarily driven by vegetation productivity and climate conditions, with soil properties unable to effectively regulate network stability due to resource limitations.

**Figure 5:**
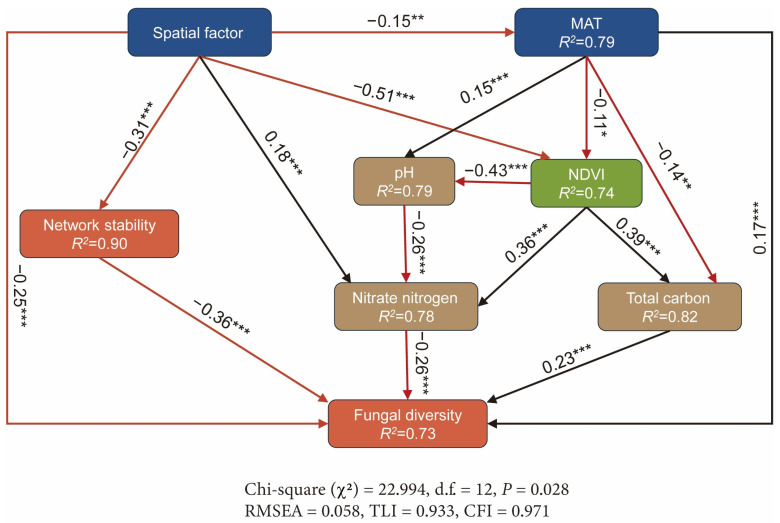
Direct and indirect pathways of environmental factors influencing soil fungal network stability and fungal community diversity. Structural equation models (SEMs) illustrate the effects of environmental factors on soil fungal network stability and diversity. Each environmental factor is associated with a path coefficient and an arrow. The strength of each relationship is represented by the path coefficient, and the arrow color indicates positive (black) or negative (red) effects. R^2^ indicates the proportion of variance explained. Statistical significance is denoted as follows: * *p* < 0.05; ** *p* < 0.01; *** *p* < 0.001. The reliability of the SEM models was confirmed through multiple statistical tests, including the chi-square test (*p* > 0.05), root mean square error of approximation (RMSEA < 0.08), Tucker–Lewis Index (TLI ≥ 0.90), and Comparative Fit Index (CFI ≥ 0.95). MAT, manual mean temperature; NDVI, normalized difference vegetation index.

In alpine shrubland habitats, the environmental factors regulating fungal community network stability show complex nonlinear relationships, with a strong model fit (adjusted *R*^2^ = 0.765, deviance explained = 96.8%). The MAT (*p* < 0.001), MAP (*p* < 0.001), NDVI (*p* = 0.014), soil pH (*p* = 0.012), NH₄^+^ (*p* < 0.001), and AP (*p* < 0.001) all have significant nonlinear positive effects on network stability. However, TN (*p* = 0.078), TC (*p* = 0.115), and NO_3_^−^ (*p* = 0.173) do not significantly affect network stability. The SEM results indicate that environmental factors (including MAT, NDVI, and soil properties) and spatial factors jointly and significantly influence network stability and fungal diversity. Overall, these factors together explain 73% of the variation in fungal α-diversity and 90% of the variation in network stability (Figure 5).

First, spatial factors significantly influence environmental conditions, showing negative effects on the MAT and NDVI (estimate = −0.15, *p* = 0.014; estimate = −0.51, *p* < 0.001, respectively). Meanwhile, the MAT negatively affects the NDVI (estimate = −0.11, *p* = 0.037). These relationships indicate that spatial patterns may indirectly regulate environmental conditions by affecting temperature and vegetation cover (Table S9).

In terms of soil properties, the MAT and NDVI significantly regulate soil pH, TC, and NO_3_^−^. The MAT positively affects soil pH (estimate = 0.15, *p* = 0.005), whereas the NDVI negatively affects soil pH (estimate = −0.43, *p* < 0.001) (Table S9). The NDVI also exerts positive effects on TC and NO_3_^−^ (estimate = 0.39, *p* < 0.001; estimate = 0.36, *p* < 0.001), indicating that higher vegetation cover enhances the availability of organic carbon and nitrogen in the soil, providing more resources to support fungal communities. In contrast, the MAT has a negative effect on TC and NO_3_^−^ (estimate = −0.14, *p* = 0.009; estimate = −0.26, *p* < 0.001), suggesting that increased temperature may reduce nutrient availability, thereby influencing resource utilization by fungal communities.

Spatial factors negatively affect network stability (estimate = −0.31, *p* < 0.001) and fungal richness (estimate = −0.25, *p* < 0.001) (Table S9). Furthermore, there is a negative relationship between network stability and fungal richness (estimate = −0.36, *p* < 0.001), indicating that higher network stability may restrict species richness expansion through mechanisms such as resource centralization.

The influence of climate factors and soil nutrients on fungal richness was also validated. The MAT positively correlates with fungal richness (estimate = 0.17, *p* = 0.001) (Figure 4, Table S9). TC also positively affects fungal richness (estimate = 0.23, *p* < 0.001), while NO_3_^−^ exerts a negative effect (estimate = −0.26, *p* < 0.001).

## 4. Discussion

This study highlights the crucial role of the NDVI and MAT in regulating fungal diversity and network functionality, demonstrating that spatial heterogeneity indirectly influences soil physicochemical properties by controlling temperature and vegetation cover, thereby shaping the coupling relationship between fungal diversity and network stability. The study integrated the combined effects of climate, vegetation cover, soil nutrients, and spatial factors at a regional scale, emphasizing the sensitivity and adaptability of fungal communities to multiple environmental changes. Furthermore, we performed large-scale network analysis and cross-habitat comparisons, particularly in resource-limited or environmentally heterogeneous ecosystems, distinguishing this study from previous research centered on fungal diversity or community composition.

### 4.1. Cross-Habitat Heterogeneity of Fungal Diversity and Community Composition

In all habitats, *Ascomycota* dominated the fungal community, followed by *Basidiomycota* (Figure 2a), consistently with global soil fungal diversity patterns [1]. *Ascomycota* demonstrates strong abilities to decompose complex organic matter, conferring significant competitive advantages in resource-scarce or environmentally vulnerable habitats [33], while *Basidiomycota* comprises root-invading taxa capable of breaking down lignin- and cellulose-rich substrates, thereby further enhancing organic matter turnover and nutrient acquisition [34].

Functional group analyses revealed that saprotrophic fungi were significantly more abundant in alpine meadows and desert steppes than in alpine shrublands, whereas the proportion of symbiotic fungi was higher in desert steppes; pathogenic fungi showed no notable differences among habitats. These distribution patterns reflect not only variations in vegetation cover and resource inputs [35], but also key climatic drivers, such as soil temperature regimes, moisture availability, and precipitation patterns, that jointly shape the functional composition of soil fungal communities [36]. Alpine meadows, characterized by moderate growing-season temperatures and annual precipitation of 400–600 mm, are dominated by herbaceous plants that supply abundant organic carbon and create heterogeneous microhabitats [37], thereby promoting saprotrophic fungal proliferation [22]. Alpine shrublands, with dense woody canopy shading, exhibit lower soil temperatures and reduced moisture retention, which limit litter decomposition and saprotrophic activity [38]. Desert steppes, in contrast, experience low annual precipitation concentrated in the summer, large diurnal temperature fluctuations, and limited soil moisture—conditions under which plants increasingly depend on symbiotic fungi to enhance water uptake and nutrient acquisition [39]. In desert steppes, where water and nutrients are scarce, plants primarily rely on symbiotic fungi to enhance water uptake and nutrient acquisition. In contrast, the distribution of pathogenic fungi is governed mainly by host plant community composition and immune defenses, rather than environmental differentiation [40]. In all three habitats, the abundance and community composition of pathogenic fungi did not differ significantly. The distribution of pathogenic fungi is typically constrained by host plant availability, rather than by any single environmental factor. Studies have shown that genera such as *Phymatotrichopsis*, *Sclerotium*, and *Athelia* often act as facultative parasites on herbaceous plants, whereas *Armillaria*, *Corticium*, and *Ganoderma* preferentially infect woody hosts [41,42]. Although our study did not directly determine the host specificity of these pathogenic genera, all three habitat types contain both herbaceous and woody plant species, likely providing similar host resources, and thus underpinning the consistent abundance and composition of pathogenic fungi across habitats. Consequently, no significant differences in pathogenic fungal communities were observed among the different habitats.

Random forest analysis identified MAT and NDVI as critical drivers for fungal richness across habitats (Figure 2d). The MAT directly influences fungal metabolic activity and interactions with plants, while the NDVI is relevant to vegetation productivity and carbon supply for soil fungi [43]. This climate–vegetation synergy aligns with the energy–environment hypothesis, highlighting temperature and resource availability as key fungal diversity drivers. Further regression analyses showed that total carbon (TC) and NO_3_^−^ both exhibited significant positive correlations with fungal richness at the regional scale (Figure S3f,h). TC provides energy for heterotrophic fungi [44], whereas NO_3_^−^ is their primary nitrogen source, and directly participates in fungal metabolism and community assembly [45]. It can thus be inferred that, in cross-habitat regions of the southwestern Tibetan Plateau, vegetation indirectly enhances fungal species richness by regulating soil organic carbon (SOC) and nitrate nitrogen (NO_3_^−^) levels, thus providing fungi with complementary energy and nitrogen resources. SOC can be utilized by fungi for respiration and ATP synthesis, supplying energy for various physiological processes [46], while NO_3_^−^ serves as the essential nitrogen source for amino acid, protein, and nucleic acid synthesis [47]. An optimal soil C:N ratio maximizes fungal carbon and nitrogen assimilation efficiency, simultaneously satisfying energy metabolism and biosynthetic demands, thereby promoting fungal growth and increasing community diversity [48]. This finding is consistent with Cui et al. [49], who reported that higher SOC and NO_3_^−^ levels significantly increased fungal α-diversity in temperate forests.

CAP analysis and partial Mantel tests further confirmed that the MAT is the strongest climatic factor driving changes in fungal β-diversity (Figure 2a, Table S1). However, its explanatory power declined substantially after controlling soil factors, indicating that climate influences fungal communities largely through modifications of soil environments. For instance, precipitation can lower soil pH via leaching, granting a competitive edge to acidophilic fungi, such as Actinomyces, and leading to shifts in fungal community structure [50]. Additionally, the reduction in fungal community similarity with increasing geographic distance [51] illustrates the combined effects of environmental heterogeneity and dispersal limitation.

The results of this study contrast with findings by Jiao et al. [51] in arid regions of northwestern China, where the aridity index (AI) exerted a greater influence on β-diversity than soil factors; in contrast, our research region—located in southwestern Tibet, with high altitude, low temperatures, and relatively fragmented hydrothermal conditions—shows that climate primarily shapes fungal distribution indirectly through vegetation cover and soil properties [52]. The soils of the southwestern Tibetan Plateau exhibit higher heterogeneity and broader pH ranges [53], contrasting dramatically with the extreme drought environment in northwestern arid areas that enforces a stronger direct dependence of fungal diversity patterns on climatic factors [54]. Meanwhile, our findings align with the work by Chen et al. [32] in the temperate grasslands of northern China, concluding that, despite variations in climate and soil conditions, plant communities remain the decisive factor governing fungal diversity and functional structure at the regional scale. In northern grasslands, abundant soil organic carbon and nitrogen support functional diversification, whereas in the Tibetan Plateau, low temperatures and hypoxic conditions drive fungi to adapt to complex environmental changes through metabolic regulation and niche differentiation [55].

### 4.2. Drivers of Fungal Network Structure

The fungal co-occurrence network exhibits a high degree of modularity (modularity = 0.879), indicating clear clustering and functional differentiation among different fungal groups within the community. This characteristic helps to maintain the functional stability of individual modules under disturbances, relying on a small number of hub and connector nodes to sustain overall connectivity and adaptive capacity [13].

In terms of functional group composition, saprotrophic fungi account for 70.91% of the total network nodes, playing a dominant role in organic matter decomposition and nutrient cycling; meanwhile, symbiotic and pathogenic fungi constitute 19.33% and 9.8%, respectively. Although their proportions are relatively small, they hold key positions in specific modules. For example, the arbuscular mycorrhizal fungus (*Glomeromycota*, ASV_1196) acts as a connector node (Figure 4b, Table S3), transferring organic carbon between plants and other microorganisms, while delivering inorganic nutrients such as phosphorus and nitrogen to plants [56], thereby enhancing resource redistribution and overall network responsiveness. Meanwhile, the Ascomycota pathogen ASV_2908 serves as a hub node within its module (Figure 4b, Table S3), indirectly regulating interspecies competition by influencing plant growth and resource allocation, thus maintaining dynamic balance in the module [57]. The complementary functions among these key nodes underscore how different fungal functional groups synergistically sustain ecological network operation, providing the whole fungal community with greater resilience and adaptive potential along environmental gradients.

Random forest analysis indicates that the MAT and MAP significantly affect the average path length of the network (Figure 4d). As the MAT rises, fungal metabolism and mycelial expansion intensify, potentially shortening resource transmission paths within the network [58]. Although the MAP indirectly affects network structure by regulating soil moisture [59], Mantel tests detected no direct correlation between the MAP and network indices (Table S4), possibly because precipitation variation in the study area is relatively small, and does not form a pronounced moisture gradient. Under relatively stable moisture conditions, vegetation cover and soil nutrient status serve as the primary drivers of network structural differences [59].

Notably, the NDVI shows a positive correlation with the node number, edge number, and mean connectivity (Table S4), indicating that higher vegetation cover can increase soil microenvironmental heterogeneity and niche complexity, thereby attracting more fungal species to participate in interactions, expanding the network, and heightening structural complexity [60]. Soil pH likewise significantly affects network structure, and its positive correlation with the node number, edge number, and average path length (Table S4) suggests that neutral-to-slightly acidic conditions facilitate fungal nutrient acquisition and utilization, in turn promoting interspecific cooperation and network connectivity [61].

Additionally, total soil nitrogen (TN) and total soil carbon (TC) both play important roles in maintaining network functionality (Table S4): TN can boost fungal metabolic activity and the intensity of community interactions, thereby increasing the node count and connectivity; and TC, as the primary energy source for heterotrophic fungi, supports the network’s basic functions by providing necessary substrates, thus enhancing connectivity and resource flow [62]. However, in the random forest model, the independent explanatory power of these two factors is relatively low (Figure 4d), aligning with the viewpoint of De Vries et al. [63]: when soil nutrients are relatively abundant and evenly distributed, TN and TC typically influence microbial network characteristics indirectly through interactions with variables such as pH or vegetation. Fierer et al. [35] noted that the effects of soil nutrients on microbial communities can be constrained by broader environmental contexts, for example, pH regulation of nutrient solubility and root activity, or the NDVI shaping litter inputs and microhabitat structure. Hence, the effects of TC and TN on fungal network structure and stability reflect more of a multifactorial coupling, rather than dominance by any single variable.

### 4.3. Spatial and Environmental Drivers of Fungal Diversity and Network Stability

The stability of the fungal network reflects the intra-community interaction intensity, nutrient flow efficiency, and overall network coordination. It serves as a crucial indicator for evaluating microbial community responses to environmental disturbances [64,65]. Our analyses revealed pronounced differences in network stability among the three habitats. Alpine meadows exhibited the highest stability, significantly higher than that of desert steppes and alpine shrublands. Stable ecological networks depend strongly on resource availability, species redundancy, and interactive structural traits within a given environment. Relatively abundant water, moderate growing-season temperatures, and higher vegetation cover (NDVI) in alpine meadows provide fungi with substantial organic carbon inputs and diverse microhabitats. These conditions foster a network with numerous nodes, high connectivity, and dense interaction intensity, enhancing both functional redundancy and species complementarity. Furthermore, GAM analysis shows that soil pH and NO_3_^−^ concentration have a nonlinear positive regulatory effect on network stability, implying that fungi maintain higher metabolic activity and collaboration under favorable pH and nitrogen availability [35,61]. Although the meadow fungal network exhibits relatively long average path lengths—which may somewhat constrain rapid resource transmission [66]—its complex structure and high redundancy ensure overall stability.

By contrast, severe water and nutrient limitations in desert steppes shrink the fungal network, leaving only a few highly stress-tolerant taxa to support the community structure. GAM results indicate that in this habitat, network stability mainly depends on the NDVI, MAT, and available phosphorus (AP) (R^2^ = 0.242), suggesting that, under resource constraints, vegetation productivity and fundamental climate conditions serve as limiting factors. Many studies of arid ecosystems have confirmed the positive effect of AP on fungal–plant interactions and hyphal growth [67]. However, due to simplified species composition, weak modularity, and few key nodes, such habitats have limited self-restoration capacity in the face of disturbances, resulting in overall lower stability. Alpine shrublands, on the other hand, exhibit a highly modular yet spatially heterogeneous network structure. Local modules often form around individual shrubs, where concentrated resource inputs create tightly coupled microenvironments; more generalist fungi spanning modules act as “bridges” to maintain connectivity. GAM analysis indicates that soil pH, NH_4_^+^, and AP exert nonlinear positive effects on network stability. Under suitable conditions, nitrogen and phosphorus inputs boost fungal metabolism and interactions; however, in the rainy season, a surge in NH_4_^+^ can disrupt modular balance, necessitating dynamic reorganization of fungal communities to sustain functional stability [67]. Therefore, although this system has some modular redundancy, its stability strongly depends on stable environmental conditions and the ability to buffer resource fluctuations.

SEM analysis further confirms that spatial heterogeneity in the study area is significantly negatively correlated with both fungal diversity and network stability. Highly heterogeneous terrains are often characterized by fragmented landscapes or variations in elevation, which lead to pronounced microclimatic differentiation and a lower MAT and NDVI [68,69]. This reduces soil resource inputs and increases the discontinuity of microhabitats, thereby constraining fungal distribution. Although the NDVI can have a positive effect on total carbon (TC) and nitrate (NO_3_^−^) [70], and the MAT can positively regulate pH [71], higher temperatures are frequently accompanied by accelerated decomposition of organic matter and nutrient loss, ultimately decreasing soil carbon storage. Moreover, fragmented topography and uneven resource allocation can intensify interspecific competition among fungi, causing local communities to exhibit a pattern of “dominant species in small, tightly clustered groups” [72], which further suppresses overall diversity and reduces network stability [73,74].

Furthermore, this study reveals a significant negative correlation between fungal species richness and network stability. This challenges the classic diversity–stability hypothesis, indicating that higher diversity does not necessarily confer greater system resilience in large-scale, highly heterogeneous environments. In particular, discontinuous resources and significant microhabitat differences can magnify negative interactions such as competition and excessive functional overlap: once species numbers increase, if there is insufficient functional redundancy or modular structure to “isolate” conflicts, negative connections tend to accumulate and spread rapidly through the network [64,74]. If critical nodes or core species are lost in a highly connected network, these adverse effects can rapidly propagate, leading to the collapse of the entire network due to reduced network stability. Although “local modules” form in alpine shrubland habitats to partially buffer disturbances, as the number of fungal ASVs increases, negative interactions not only accumulate within each module, but also spread throughout the network via cross-module connectors. Consequently, the original stabilizing benefits of modularity are weakened: under limited resources and environmental heterogeneity, higher species diversity brings more potential competition and conflict. Once key nodes are compromised or resource availability becomes unbalanced, chain reactions across modules can rapidly affect the entire network, causing overall stability to decrease as diversity rises.

In summary, fungal networks in alpine meadows, desert steppes, and alpine shrublands exhibit marked differences in structure and stability. Furthermore, under large-scale, highly heterogeneous conditions, higher species richness does not necessarily correlate with higher network stability. Alpine meadows remain robust thanks to ample water, moderate temperatures, and high vegetation cover; desert steppes, conversely, suffer from severe aridity and limited nutrient availability, resulting in simplified networks and low stability; while alpine shrublands, despite pronounced modularity, struggle with exacerbated negative interactions as community diversity rises. These outcomes highlight spatial heterogeneity, vegetation productivity, and soil properties collectively shape fungal communities, revealing a more intricate balance between diversity and stability and offering valuable insights for understanding and managing biodiversity in high-altitude ecosystems.

## 5. Conclusions

This study examined how spatial and environmental factors influence the geographical distribution, diversity, and network structure of soil fungal communities in the southwestern Tibetan Plateau. The results show that Ascomycota dominates across habitats, with notable community and diversity differences. Saprotrophic fungi are more abundant in alpine meadows and desert steppes, while symbiotic fungi occur more frequently in desert steppes; pathogenic fungi show no significant differences among habitats. Fungal α-diversity is significantly higher in alpine meadows and desert steppes than in alpine shrublands. The MAT and NDVI are key drivers of α-diversity, whereas soil variables primarily control β-diversity. A highly modular fungal ecological network was identified: the NDVI notably boosts network size and connectivity by increasing resource inputs, and soil pH influences network stability through module distribution. SEM revealed that spatial factors directly affect fungal diversity and indirectly shape community and network structures by mediating soil properties via the MAT and NDVI. Furthermore, spatial heterogeneity limits network stability and diversity through uneven resource distribution and niche segregation. This study reveals how spatial, climatic, and soil factors interplay within fungal ecological networks, deepening our understanding of fungal diversity patterns. Following this study, future work should track dynamic shifts in fungal functional groups under changing climate and soil conditions.

## Data Availability

Raw sequencing data and metadata supporting the results have been deposited in Figshare (https://doi.org/10.6084/m9.figshare.28256123, accessed on 6 May 2025).

## Supplementary Materials

The following supporting information can be downloaded at https://www.mdpi.com/article/10.3390/jof11050389/s1. Figure S1: Soil fungal functional groups across different habitats; Figure S2: Correlation between soil fungal richness and environmental factors; Figure S3: Overall patterns of soil fungal β-diversity across different habitats; Figure S4: Network topology characteristics across different habitats; Figure S5: Relationship between soil fungal network stability and environmental factors; Table S1: ANOVA of environmental factors correlated with soil fungal β-diversity; Table S2: ANOVA of environmental factors correlated with soil fungal β-diversity in alpine meadows; Table S3: ANOVA of environmental factors correlated with soil fungal β-diversity in desert steppe habitats; Table S4: ANOVA of environmental factors correlated with soil fungal β-diversity in shrubland habitats; Table S5: Mantel and partial Mantel tests for correlations between fungal β-diversity and edaphic and climatic factors; Table S6: Topological roles, taxonomic classification, and trophic mode of fungal ASVs; Table S7: Correlations of network structure with environment factors, as revealed by Mantel test; Table S8: Generalized Additive Model (GAM) results for soil fungal network stability across different habitats; Table S9: Path coefficients of structural equation model (SEM) for alpine meadows, showing relationships among variables.
