# Supplementary material for: Soil Fungal Diversity, Community Structure, and Network Stability in the Southwestern Tibetan Plateau
Source: J Fungi (Basel). 2025 May 19;11(5):389. doi: 10.3390/jof11050389 (PMC12113623; doi:10.3390/jof11050389)
Supplement: Supplementary file 1 [file jof-11-00389-s001.zip › jof-3608208-supplementary.pdf]

Supplementary Information

Soil Fungal Diversity, Community Structure and Network Stability in Southwestern Tibetan Plateau

Shiqi Zhang [1,2], Zhenjiao Cao [1,2], Siyi Liu [1], Zhipeng Hao [1], Xin Zhang [1,2], Guoxin Sun [1,2], Yuan Ge [1,2], Limei Zhang [1,2] and Baodong Chen [1,2,*]

[1] State Key Laboratory of Regional and Urban Ecology, Research Center for Eco-Environmental Sciences,
Chinese Academy of Sciences, Beijing 100085, China; sqzhang_st@rcees.ac.cn (S.Z.);
caozhenjiao@ibcas.ac.cn (Z.C.); syliu@rcees.ac.cn (S.L.); zphao@rcees.ac.cn (Z.H.);
xinzhang@rcees.ac.cn (X.Z.); gxsun@rcees.ac.cn (G.S.); yuange@rcees.ac.cn (Y.G.);
zhanglm@rcees.ac.cn (L.Z.)

[2] University of Chinese Academy of Sciences, Beijing 100049, China

* Correspondence: bdchen@rcees.ac.cn; Tel.: +86-010-6284-9068

The supplementary materials contain 5 figures and 9 tables.

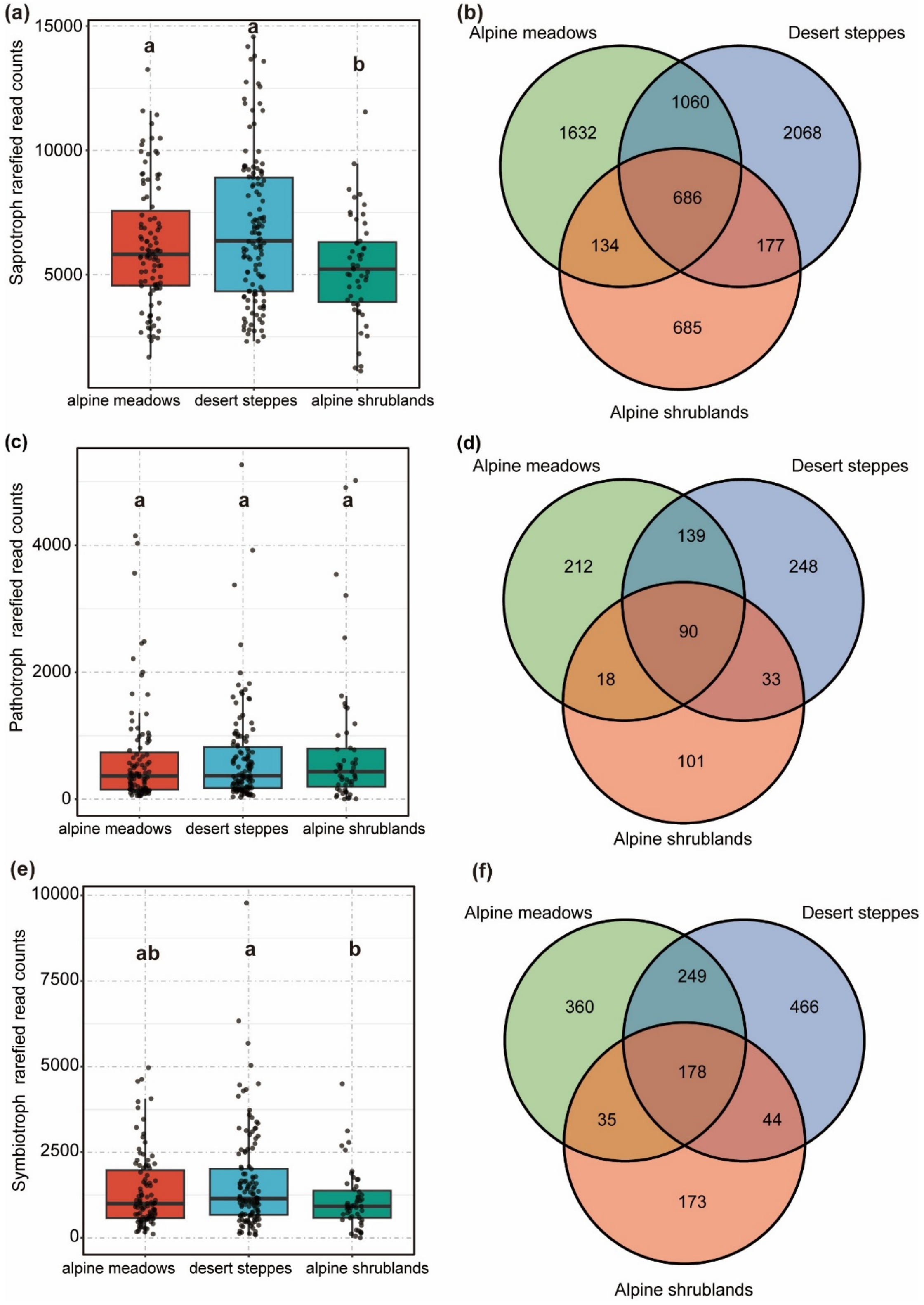

Fig S1. Soil fungal functional groups across different habitats. (a, c, e) show the differences in rare reads (ASV abundance) of saprotrophic, pathogenic, and symbiotic fungi in different habitats, such as alpine meadow, desert steppe, and shrubland; (b, d, f) are Venn diagrams illustrating the unique and shared ASV numbers of saprotrophic, pathogenic, and symbiotic fungi across different habitats.

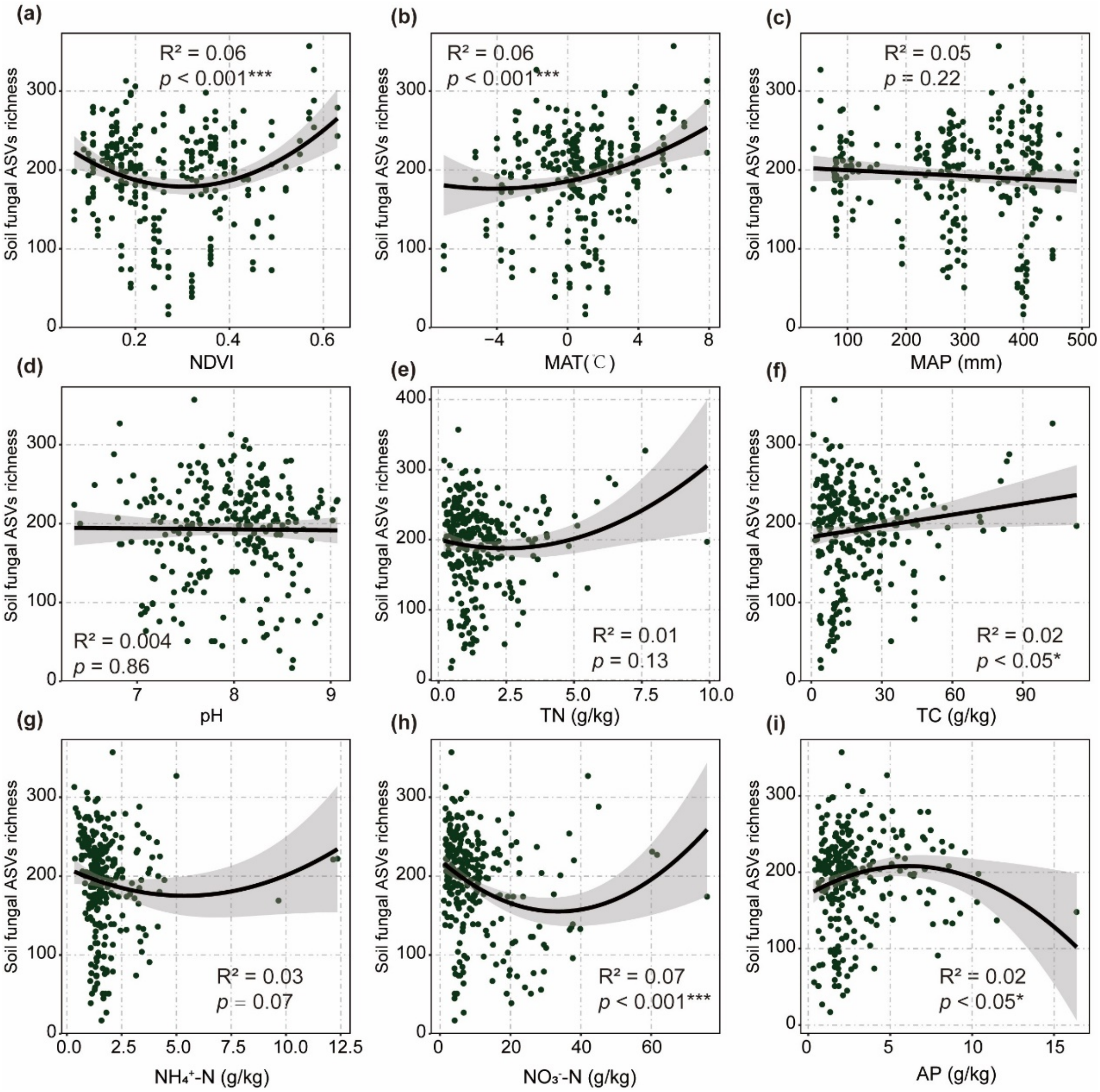

Fig S2. Correlation between soil fungal richness and environmental factors. The relationships between soil fungal richness and (a) NDVI, (b) MAT, (c) MAP, (d) pH, (e) TN, (f) TC, (g) NH_4^+, (h) NO_3^-, and (i) AP. MAT, mean annual temperature; MAP, mean annual precipitation; NDVI, normalized difference vegetation index; TN, soil total nitrogen content; TC, soil total carbon content; NH_4, soil ammonium-nitrogen content; NO_3, soil nitrate-nitrogen content; AP, soil available phosphorus content. Statistical significance: *, $p < 0.05$; **, $p < 0.01$; ***, $p < 0.001$.

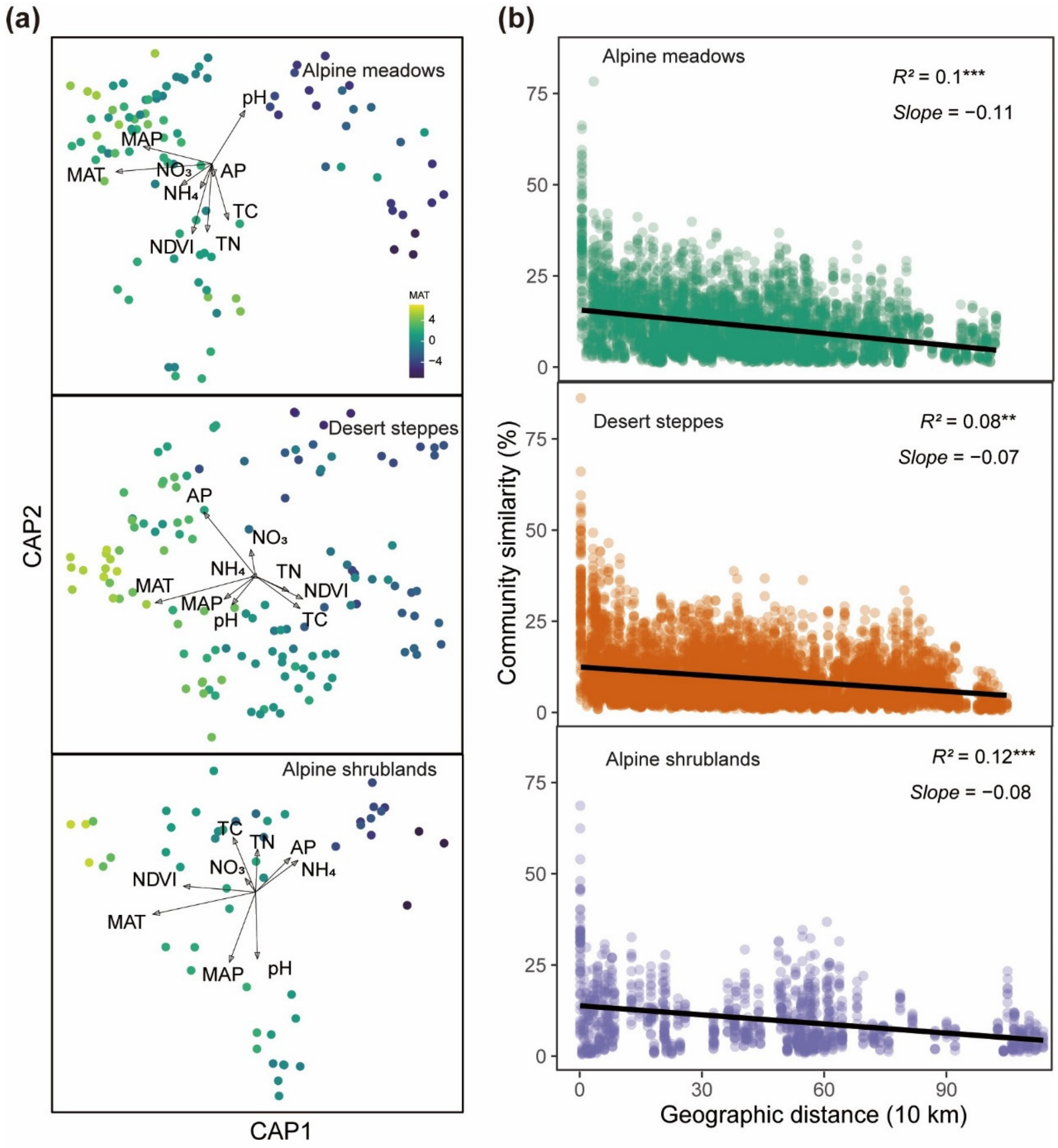

Fig S3. Overall patterns of soil fungal β-diversity across different habitats. (a) Constrained analysis of principal coordinates (CAP) showing the influence of environmental factors on fungal community structure. Sample points are colored according to MAT (mean annual temperature). (b) Distance decay curves showing Bray-Curtis similarity as a function of geographic distance between sampling points. Solid lines represent ordinary least squares linear regression. Asterisks indicate significant correlations (*, $p < 0.05$; **, $p < 0.01$; ***, $p < 0.001$).

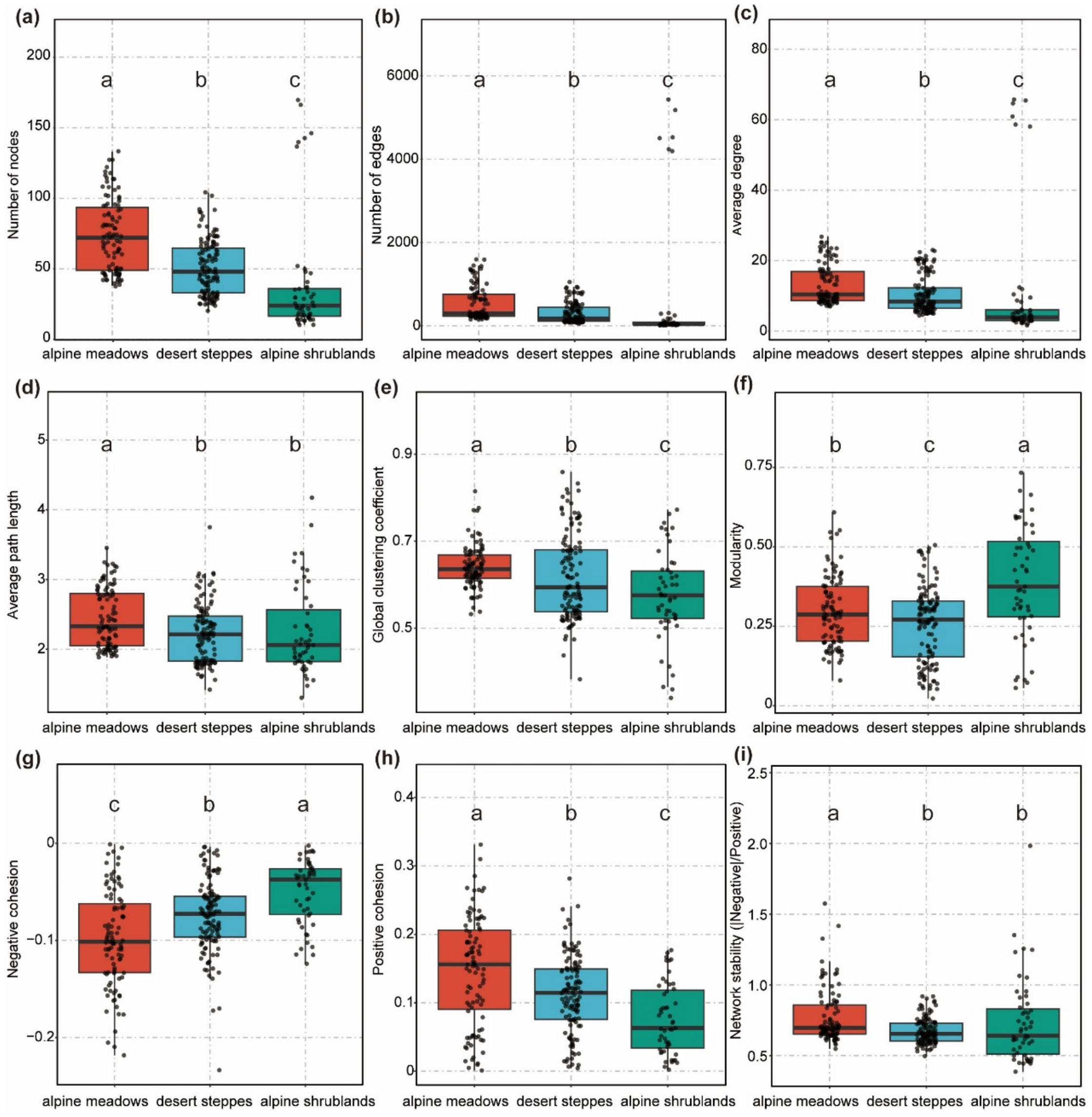

Fig S4. Network topology characteristics across different habitats. Comparison of the number of nodes (a), edges (b), and average degree (c), average path length (d), global clustering coefficient (e), and modularity (f), negative cohesion (g), positive cohesion (h), and network stability (i) in soil fungal co-occurrence networks among alpine meadows, desert steppes, and shrublands.

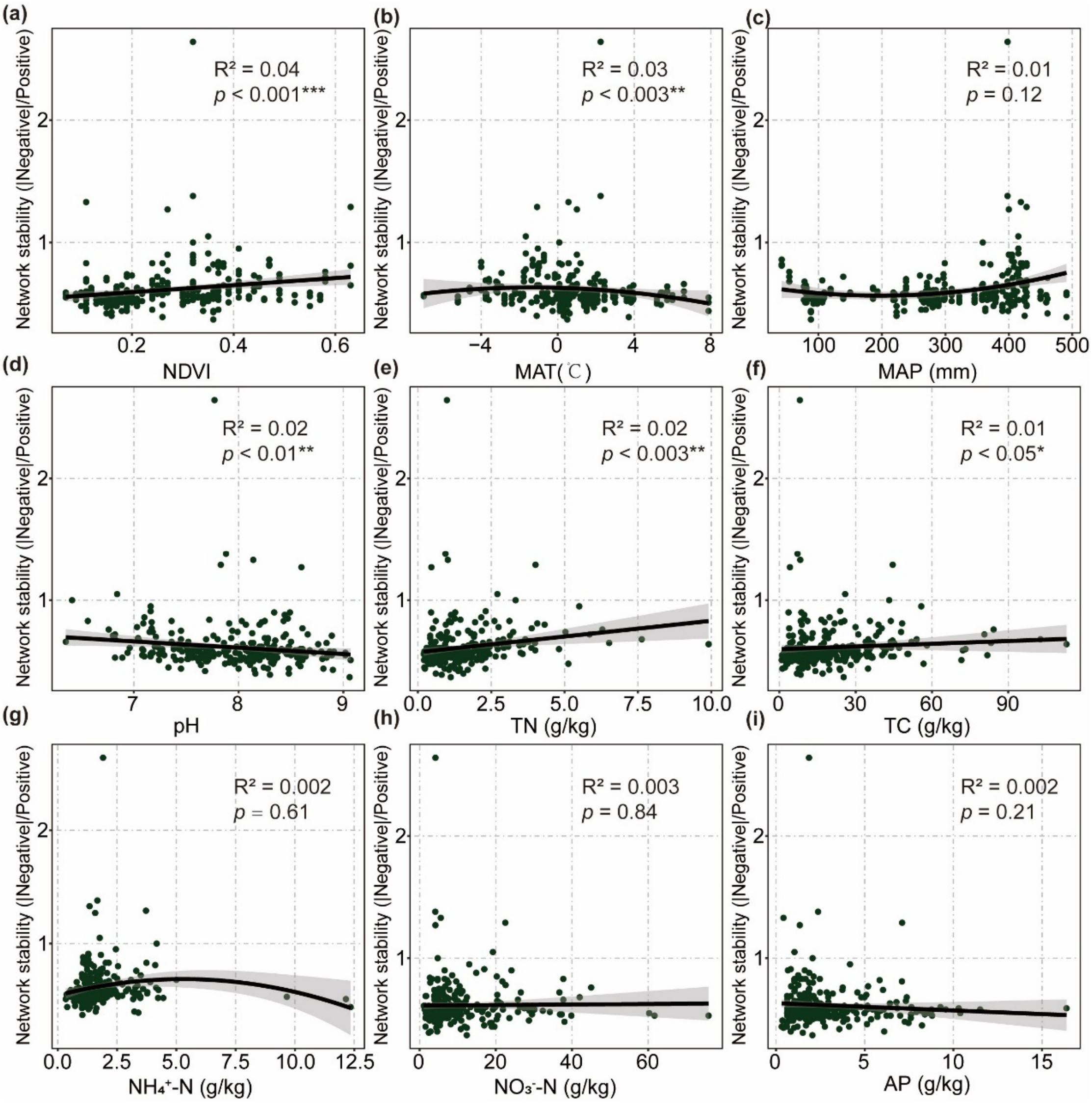

Fig S5. Relationship between soil fungal network stability and environmental factors. The relationships between network stability and (a) NDVI, (b) MAT, (c) MAP, (d) pH, (e) TN, (f) TC, (g) NH_4^+, (h) NO_3^-, and (i) AP. Network stability is calculated as the absolute difference between negative and positive cohesion. MAT, mean annual temperature; MAP, mean annual precipitation; NDVI, normalized difference vegetation index; TN, soil total nitrogen content; TC, soil total carbon content; NH_4, soil ammonium-nitrogen content; NO_3, soil nitrate-nitrogen content; AP, soil available phosphorus content. Statistical significance: *, $p < 0.05$; **, $p < 0.01$; ***, $p < 0.001$.

Table S1 ANOVA of environmental factors correlated with soil fungal *β*-diversity.

Factor	df	Variance	Pseudo-F	p-value
MAT	1	3.148	6.577	0.001
MAP	1	1.581	3.303	0.001
AP	1	1.106	2.310	0.001
pH	1	1.088	2.274	0.001
TN	1	1.046	2.184	0.001
TC	1	0.920	1.923	0.001
NDVI	1	0.830	1.734	0.001
NO_3^-	1	0.808	1.688	0.001
NH_4^+	1	0.777	1.623	0.001
Residual	266	127.325		
**Factor**	**df**	**Variance**	**Pseudo-F**	**p-value**
CAP1	1	3.621	7.564	0.001
CAP2	1	1.67	3.489	0.001
CAP3	1	1.485	3.101	0.001
CAP4	1	1.093	2.283	0.001
CAP5	1	0.836	1.746	0.001
CAP6	1	0.719	1.502	0.005
CAP7	1	0.668	1.396	0.012
CAP8	1	0.653	1.365	0.012
CAP9	1	0.56	1.171	0.05
Residual	266	127.325		

Notes. df = degree of freedom; Permutations = 999; Overall model significance: p = 0.001.

Table S2 ANOVA of environmental factors correlated with soil fungal *β*-diversity in alpine meadows.

**Factor**	**df**	**Variance**	**Pseudo-F**	**p-value**
MAT	1	1.346	3.443	0.001
pH	1	1.077	2.757	0.001
MAP	1	0.979	2.505	0.001
NDVI	1	0.899	2.300	0.001
NH_4^+	1	0.722	1.848	0.001
NO_3^-	1	0.716	1.833	0.001
TC	1	0.670	1.714	0.003
TN	1	0.641	1.641	0.002
AP	1	0.622	1.592	0.002
Residual	89	34.776		
**Factor**	**df**	**Variance**	**Pseudo-F**	**p-value**
CAP1	1	1.737	4.445	0.001
CAP2	1	1.405	3.597	0.001
CAP3	1	0.938	2.399	0.001
CAP4	1	0.884	2.262	0.001
CAP5	1	0.652	1.669	0.038
CAP6	1	0.614	1.571	0.038
CAP7	1	0.568	1.454	0.055
CAP8	1	0.467	1.195	0.258
CAP9	1	0.407	1.042	0.318
Residual	89	34.776		

Notes. df = degree of freedom; Permutations = 999; Overall model significance: p = 0.001.

Table S3 ANOVA of environmental factors correlated with soil fungal *β*-diversity in desert steppe habitats.

**Factor**	**df**	**Variance**	**Pseudo-F**	**p-value**
MAT	1	2.061	4.950	0.001
MAP	1	1.279	3.073	0.001
AP	1	1.294	3.107	0.001
TC	1	0.936	2.248	0.001
pH	1	0.913	2.193	0.001
NO_3^-	1	0.792	1.902	0.001
NDVI	1	0.687	1.650	0.001
TN	1	0.651	1.564	0.004
NH_4^+	1	0.560	1.346	0.019
Residual	116	48.290		
**Factor**	**df**	**Variance**	**Pseudo-F**	**p-value**
CAP1	1	2.297	5.518	0.001
CAP2	1	1.477	3.549	0.001
CAP3	1	1.327	3.188	0.001
CAP4	1	0.892	2.143	0.001
CAP5	1	0.744	1.787	0.003
CAP6	1	0.677	1.626	0.011
CAP7	1	0.641	1.541	0.011
CAP8	1	0.617	1.482	0.011
CAP9	1	0.499	1.198	0.062
Residual	116	48.290		

Notes. df = degree of freedom; Permutations = 999; Overall model significance: p = 0.001.

Table S4 ANOVA of environmental factors correlated with soil fungal *β*-diversity in shrubland habitats.

Factor	df	Variance	Pseudo-F	p-value
MAT	1	1.406	3.786	0.001
pH	1	0.893	2.406	0.001
NO_3^-	1	0.782	2.105	0.001
MAP	1	0.762	2.053	0.001
AP	1	0.727	1.957	0.001
TN	1	0.677	1.823	0.001
TC	1	0.665	1.791	0.001
NH_4^+	1	0.628	1.691	0.001
NDVI	1	0.609	1.641	0.002
Residual	41	15.227		
**Factor**	**df**	**Variance**	**Pseudo-F**	**p-value**
CAP1	1	2.297	5.518	0.001
CAP2	1	1.477	3.549	0.001
CAP3	1	1.327	3.188	0.001
CAP4	1	0.892	2.143	0.001
CAP5	1	0.744	1.787	0.003
CAP6	1	0.677	1.626	0.011
CAP7	1	0.641	1.541	0.011
CAP8	1	0.617	1.482	0.011
CAP9	1	0.499	1.198	0.062
Residual	116	48.290		

Notes. df = degree of freedom; Permutations = 999; Overall model significance: p = 0.001.

Table S5 Mantel and partial Mantel tests for correlations between fungal *β*-diversity and edaphic and climatic factors.

Effect	Controlling for	All habitat	Alpine meadows	Desert steppes	Shrublands
Edaphic		0.221**	0.250**	0.362**	0.257**
Climatic		0.081**	0.227**	0.190**	0.225**
Edaphic	Climatic	0.207**	0.189**	0.321**	0.171**
Climatic	Edaphic	0.017*	0.156**	0.073*	0.115*

Notes. Significance was tested using 999 permutations; * $p < 0.05$, **: $p < 0.01$, ***: $p < 0.001$.

Table S6. Topological roles, taxonomic classification and trophic mode of fungal ASVs.

ID	Zi	Pi	Type	Phylum	Trophic mode
ASV_75	3.48	0.00	Module Hub	*Ascomycota*	Saprotroph
ASV_209	2.80	0.04	Module Hub	*Ascomycota*	Symbiotroph
ASV_408	-0.58	0.67	Connector	*Ascomycota*	Saprotroph
ASV_1196	-0.96	0.63	Connector	*Glomeromycota*	Symbiotroph
ASV_1679	2.52	0.01	Module Hub	*Mucoromycota*	Saprotroph
ASV_1834	2.52	0.01	Module Hub	*Ascomycota*	Saprotroph
ASV_2009	-0.96	0.63	Connector	*Ascomycota*	Symbiotroph
ASV_2218	2.82	0.00	Module Hub	*Ascomycota*	Saprotroph
ASV_2251	2.67	0.00	Module Hub	*Ascomycota*	Saprotroph
ASV_2574	-1.75	0.67	Connector	*Ascomycota*	Saprotroph
ASV_2611	-0.96	0.63	Connector	*Ascomycota*	Symbiotroph
ASV_2875	-1.86	0.67	Connector	*Ascomycota*	Saprotroph
ASV_2908	2.75	0.47	Module Hub	*Ascomycota*	Pathotroph
ASV_3120	3.14	0.00	Module Hub	*Ascomycota*	Saprotroph
ASV_3469	2.52	0.01	Module Hub	*Ascomycota*	Saprotroph
ASV_3775	-1.46	0.67	Connector	*Ascomycota*	Pathotroph
ASV_3798	2.52	0.01	Module Hub	*Ascomycota*	Saprotroph
ASV_3990	3.02	0.47	Module Hub	*Ascomycota*	Pathotroph
ASV_4719	3.35	0.41	Module Hub	*Ascomycota*	Saprotroph
ASV_5724	2.62	0.03	Module Hub	*Ascomycota*	Pathotroph
ASV_6655	2.73	0.41	Module Hub	*Mortierellomycota*	Saprotroph
ASV_9671	3.23	0.46	Module Hub	*Basidiomycota*	Saprotroph

Table S7. Correlations of network structure with environment factors as revealed by Mantel test.

	Nodes		Edges		Average degree		Average path length		Modularity	
	r	p	r	p	r	p	r	p	r	p
NDVI	**0.13**	**0.00**	**0.27**	**0**	**0.19**	**0.00**	**0.07**	**0.01**	-0.04	0.98
MAT	**0.08**	**0.01**	**0.08**	**0.04**	-0.01	0.58	0.05	0.07	-0.05	0.98
MAP	-0.03	0.91	0.02	0.23	0.01	0.32	0.03	0.14	0.01	0.33
pH	**0.06**	**0.02**	**0.06**	**0.04**	**0.08**	**0.01**	**0.09**	**0**	0	0.51
TN	**0.09**	**0.02**	**0.19**	**0.01**	**0.14**	**0.01**	**0.09**	**0.02**	-0.07	1
TC	**0.07**	**0.05**	**0.14**	**0.02**	**0.1**	**0.02**	**0.1**	**0.01**	-0.02	0.68
NH_4^+	0	0.48	0.02	0.24	-0.02	0.68	0.01	0.32	-0.07	1
NO_3^-	0.02	0.29	0.07	0.07	0.07	0.06	-0.03	0.8	0.01	0.33
AP	-0.01	0.63	-0.01	0.55	-0.01	0.61	-0.02	0.7	0	0.49

Significant correlations are highlighted in bold.

Table S8. Generalized Additive Model (GAM) results for soil fungal network stability across different habitats.

	Alpine meadow				Desert steppe				Shrubland			
	edf	Ref.df	F-value	p-value	edf	Ref.df	F-value	p-value	edf	Ref.df	F-value	p-value
NDVI	5.869	6.793	6.470	***	1.000	1.000	2.331	0.130	7.079	7.328	17.536	***
MAT	3.108	3.719	9.925	***	1.000	1.000	3.990	**0.048**	1.000	1.000	14.104	***
MAP	1.000	1.000	0.471	0.495	1.537	1.881	10.699	**0.001**	1.000	1.000	1.117	0.292
pH	3.659	4.519	10.241	***	1.000	1.000	3.738	0.056	7.268	7.573	8.809	***
TN	3.337	4.105	1.194	0.315	1.000	1.000	0.001	0.980	6.477	6.685	7.923	***
TC	2.693	3.304	1.650	0.201	1.000	1.000	0.216	0.643	7.084	7.311	18.898	***
NH_4^+	1.376	1.598	5.453	0.052	1.000	1.000	1.507	0.222	5.885	6.147	6.897	***
NO_3^-	2.158	2.610	5.672	**0.004**	1.000	1.000	2.491	0.117	6.758	7.061	5.215	***
AP	1.000	1.000	0.670	0.416	1.000	1.000	9.477	**0.003**	2.194	2.324	1.690	0.104
Adjusted R^2	0.736				0.242				0.959			
Deviance explained	80.1%				29.9%				96.8%			

Notes: This table presents the estimated degrees of freedom (edf), reference degrees of freedom (Ref.df), F-values, and p-values for the effects of environmental variables on soil fungal network stability in alpine meadow, desert steppe, and shrubland. The analyzed predictors include Normalized Difference Vegetation Index (NDVI), Mean Annual Temperature (MAT), Mean Annual Precipitation (MAP), Soil pH, Total Nitrogen (TN), Total Carbon (TC), Ammonium Nitrogen (NH_4^+), Nitrate Nitrogen (NO_3^-), and Available Phosphorus (AP). Significant effects are indicated by *** (p < 0.001).

Table S9. Path coefficients of the structural equation model (SEM) for alpine meadows, showing relationships among variables.

Factor	Estimate	S.E.	C.R.	P level
MAT<---Special factor	-0.15	0.06	-2.46	0.014
NDVI<---Special factor	-0.51	0.05	-9.75	***
NDVI<---MAT	-0.11	0.05	-2.08	0.037
pH<---NDVI	-0.43	0.05	-7.97	***
pH<---MAT	0.15	0.05	2.79	0.005
TC<---NDVI	0.39	0.06	7.13	***
NO_3^-<---NDVI	0.36	0.07	5.35	***
TC<---MAT	-0.14	0.06	-2.63	0.009
NO_3^-<---pH	-0.26	0.06	-4.40	***
cohesion<---Special factor	-0.31	0.06	-5.40	***
NO_3^-<---Special factor	0.18	0.06	2.91	0.004
Richness<---cohesion	-0.36	0.05	-6.56	***
Richness<---TC	0.23	0.05	4.19	***
Richness<---Special factor	-0.25	0.06	-4.41	***
Richness<---MAT	0.17	0.05	3.22	0.001
Richness<---NO_3^-	-0.26	0.05	-4.91	***

Notes: This table lists the estimated path coefficients (Estimate), standard errors (S.E.), critical ratios (C.R.), and significance levels (P values). Significant paths are indicated as follows: * $p < 0.05$, **: $p < 0.01$, ***: $p < 0.001$.
